# Characteristics of in-hospital mortality of congenital heart disease (CHD) after surgical treatment in children from 2005 to 2017: a single-center experience

**DOI:** 10.1186/s12887-021-02935-2

**Published:** 2021-11-23

**Authors:** Guilang Zheng, Jiaxing Wu, Peiling Chen, Yan Hu, Huiqiong Zhang, Jing Wang, Hanshi Zeng, Xufeng Li, Yueyu Sun, Gang Xu, Shusheng Wen, Jianzheng Cen, Jimei Chen, Yuxiong Guo, Jian Zhuang

**Affiliations:** 1 Pediatric Intensive Care Unit, Department of Pediatrics, Guangdong Provincial People’s Hospital (GDPH), Guangdong Academy of Medical Sciences, Guangzhou, China; 2 Department of Cardiac Surgery, Guangdong Cardiovascular Institute, Guangdong Provincial People’s Hospital (GDPH), Guangdong Academy of Medical Sciences, 106 zhongshan Er Road, Guangzhou, Guangdong China

**Keywords:** Congenital heart disease, Epidemiology, In-hospital mortality, Pediatric cardiac surgery

## Abstract

**Background:**

To evaluate trends in the in-hospital mortality rate for pediatric cardiac surgery procedures between 2005 and 2017 in our center, and to discuss the mortality characteristics of children’s CHD after thoracotomy.

**Methods:**

This retrospective data were collected from medical records of children underwent CHD surgery between 2005 and 2017.

**Results:**

A total of 19,114 children with CHD underwent surgery and 444 children died, with the in-hospital mortality was 2.3%. Complex mixed defect CHD had the highest fatality rate (8.63%), left obstructive lesion CHD had the second highest fatality rate (4.49%), right to left shunt CHD had the third highest mortality rate (3.51%), left to right shunt CHD had the lowest mortality rate (χ2 = 520.3,*P* < 0.05). The neonatal period has the highest mortality rate (12.17%), followed by infant mortality (2.58%), toddler age mortality (1.16%), and preschool age mortality (0.94%), the school age and adolescent mortality rate was the lowest (χ2 = 529.3,*P* < 0.05). In addition, the fatality rate in boys was significantly higher than that in girls (2.77% versus 1.62%, χ2 = 26.4, *P* < 0.05).

**Conclusions:**

The mortality rate of CHD surgery in children decreased year by year. The younger the age and the more complicated the cyanotic heart disease, the higher the mortality rate may be.

## Background

Congenital heart disease (CHD), which accounts for approximately 1/3 of common birth defects, is one of the most common abnormalities in infants, with an incidence of 1–22 per 1000 live births in China [1]. In the USA, the incidence of CHD in premature infants, excluding isolated patent ductus arteriosus (PDA) and atrial septal defect (ASD), is 12.5 per 1000 live births [2]. Despite advances in detection and treatment, CHD accounts for 3% of all infant deaths and 46% of deaths from congenital malformations [3]. Without surgery, children with CHD are often incompatible with long-term survival. Prognosis and quality of life have considerably improved in patients with CHD over the past few decades, mainly due to earlier diagnosis and ongoing improvement of surgical and anesthesia technology performed in this population [4]. At present, there are many reports on the mortality rate of congenital heart disease, but there are few reports focusing on the chronological changes in the mortality rate in China and the changes in the mortality rates among children at different ages, in different years, of different sexes and with different heart disease types [5–9]***.***

This study aimed to evaluate trends in the in-hospital mortality rate for pediatric cardiac surgery procedures between 2005 and 2017 in our center and to discuss the mortality characteristics of children’s CHD after thoracotomy.

## Methods

We reviewed congenital heart defects in children in our hospital during the 13 years between January 2005 and December 2017. Clinical data were collected from the hospital database and extracted from the medical records by the Medical Records Section at our hospital. The main variable analyzed was in-hospital mortality and the distribution of mortality by disease type, age and sex was evaluated. This study was a retrospective study and did not involve any risk to the children’s privacy. Moreover, this epidemiological investigation did not pose any harm to the patients. Therefore, permission was obtained from our unit ethics committee.

A preliminary diagnosis of CHD can be obtained in combination with the patient’s symptoms and signs. Confirmation of the diagnosis mainly relies on cardiac color ultrasound and CT scans of the heart and large vessels. Complicated CHD preoperative evaluation sometimes chooses heart 3D printing or pulmonary angiography to assist in the selection of surgical options. Patients with severely impaired myocardial function sometimes choose cardiac MRI for preoperative evaluation. Through the above diagnostic procedures, all patients were diagnosed in our hospital. And the inclusion criteria were:①Children with CHD who were hospitalized for surgery,②Children with thoracotomy excluding interventional surgery,③Because CHD operations were scattered in different cardiac surgery departments for children over 14 years, and our focus was on children under 14, so this study only included children younger than 14 years old. Non-CHD patients, non-thoracotomy children, or age-inconsistent patients were excluded.

After excluding 56 cases that did not meet the study requirements, a total of 19,144 children were finally included in this retrospective study. All children were younger than 14 years old, including 11,751 boys and 7363 girls. According to the characteristics of child growth and development, the children were divided into 5 groups according to their age: neonatal period, infant period, toddler age, preschool age and school age and adolescence. The first 28 days were considered the neonatal period, while the first year of life was the infant period. Toddler age referred to the second and third years after birth. The preschool age group included children aged 3 to 6 years, and children between 6 and 14 years of age were included in the school age and adolescent group.

The International Classification of Diseases, Ninth Revision (ICD-9) was used for the classification of CHD. We divided all CHDs of children into five categories [10, 11]: ① left to right shunt CHD, ② left obstructive lesion CHD, ③ right to left shunt CHD, ④ complex mixed defect CHD, and ⑤ other CHD (Table 1)**.**

**Table 1:** The pathophysiology classification of CHD

Non-cyanotic CHD	Cyanotic CHD
①**Left to right shunt CHD**	③**Right to left shunt CHD**
Ventricular septal defect (VSD)	Tetralogy of Fallot (TOF)
Atrial septal defect (ASD)	Pulmonary stenosis (PS)
Patent ductus arteriosus (PDA)	Pulmonary atresia (PA)
Atrioventricular septal defect (AVSD)	Tricuspid atresia
Aortopulmonary window	Ebstein deformity
②**Left obstructive lesion CHD**	Double outlet right ventricle (DORV)
Coarctation of the aorta (COA)	④**Complex mixed defect CHD**
Interrupted or hypoplastic aortic arch (I/HAA)	Transposition of the great arteries (TGA)
Aortic stenosis (AS)	Totally anomalous pulmonary venous drainage (TAPVD)
Mitral valve lesion (MV)	Permanent arterial stem (PAT)
⑤**Other CHD**	Left ventricular dysplasia syndrome
Infective endocarditis, Cardiac tumor, Coronary origin abnormality, Congenital pulmonary sling	

Statistical analyses were performed using SPSS (Statistical Package for the Social Sciences) version 19.0 software (SPSS Inc). Categorical variables are presented as numbers and percentages. Comparisons between groups were performed for the distribution of categorical variables using the chi-squared test, and z-test is used for comparison between groups, while Bonferroni method using for adjust *p*-values. A two-sided *p*-value of < 0.05 was considered indicative of statistical significance.

## Results

**Fig. 1:**
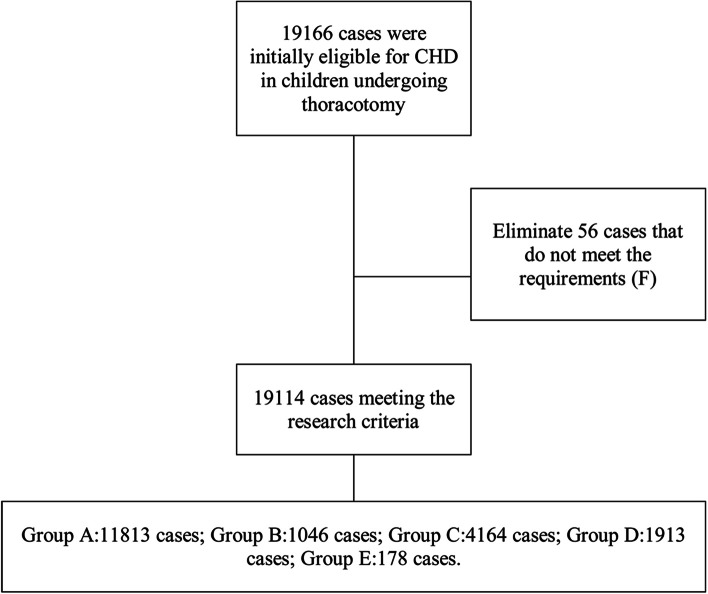
Patient flow chart. Group A: left to right shunt CHD; Group B: left obstructive lesion CHD; Group C: right to left shunt CHD; Group D: complex mixed defect CHD; Group E: other CHD. Reasons for exclusion (F) were portal vein-hepatic artery fistula (1 case), atrial benign tumor (8 cases), infectious endocarditis with neoplasms (32 cases), prolonged (chronic) myocarditis (1 case), persistent fetal circulation (1 case), constrictive pericarditis (1 case), atrial thrombus (1 case), congenital paralysis (1 case), ascending aortic aneurysm (1 case), endometrial fibrosis (1 case), third-degree atrioventricular block (1 case), explosive myocarditis (2 cases), and mediastinal benign tumors (1 case)

There were 19,166 cases of CHD in children undergoing thoracotomy at our hospital during the study period (2005 to 2017) who were initially eligible. Of those, 56 cases that did not meet the inclusion criteria were eliminated. A total of 19,114 cases meeting the research criteria were selected to participate in the study. Most of the patients (59.6%) were male. A total of 11,813 children had left to right shunt CHD (in group A), 1046 cases had left obstructive lesion CHD (in group B), 4164 cases had right to left shunt CHD (in group C), 1913 cases had complex mixed defect CHD (in group D), and 178 cases had other CHD (in group E) (Fig. 1). All children were under 14 years old. The age composition was 1060 (5.5%) newborns, 8447 (44.1%) infants under 1 year old, 4040 (21.1%) toddler age children, 3192 (16.7%) preschool age children, and 2375 (12.4%) school age children and adolescents. Most patients were under 3 years old (70.8%).

**Fig. 2:**
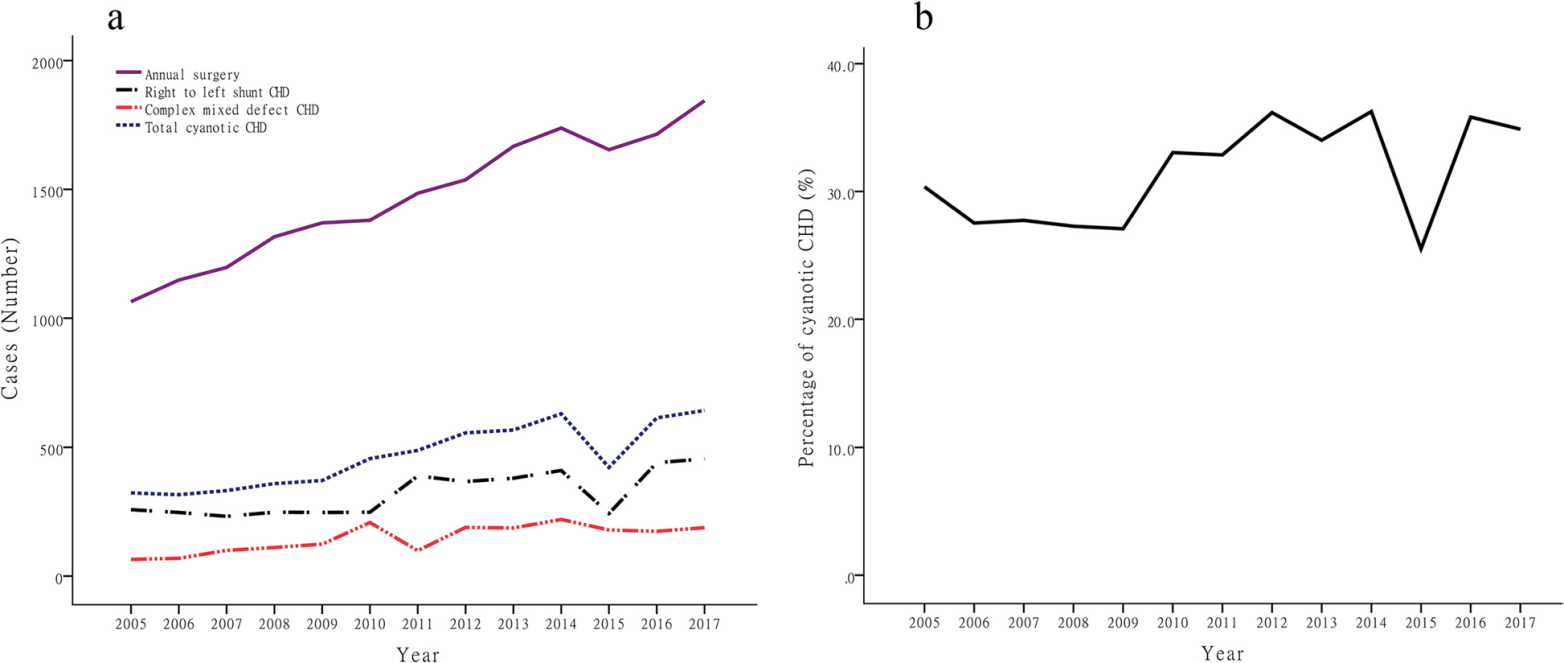
Annual operation volume for CHD and the cyanotic CHD proportion in children below 14 years old from 2005 to 2017. Chart **a** shows the annual cases of surgery for right-to-left shunt CHD, complex mixed defect CHD and total cyanotic CHD from 2005 to 2017. Chart **b** demonstrates the cyanotic CHD proportion (of total CHD in children below 14 years old) from 2005 to 2017

From 2005 to 2017, the total amount of CHD surgery in our hospital continued to increase. The annual surgical volume increased from 1064 cases in 2005 to 1844 cases in 2017, with an annual surgical growth rate of 73.3%. The annual increase in the number of CHD operations fluctuated from 49 to 133, but the amount of surgery decreased slightly in 2015. In this article, cyanotic CHD was composed of both complex mixed defect CHD and right to left shunt CHD. The annual surgical volume of cyanotic CHD increased from 323 in 2005 to 643 in 2017, with an annual surgical growth rate of 99.1%. Although there was sometimes a slight dip, the proportion of cyanosis CHD gradually increased, from 30.4% in 2005 to 34.9% in 2014. Similar to annual surgery, the volume of right to left shunt CHD and complex mixed defect CHD increased gradually. The former increased from 258 in 2005 to 455 in 2017, and the latter increased from 65 in 2005 to 188 in 2017. In 2015, both the total amount of CHD surgery and the percentage of cyanotic CHD showed a sudden and significant decline (Fig. 2).

**Fig. 3:**
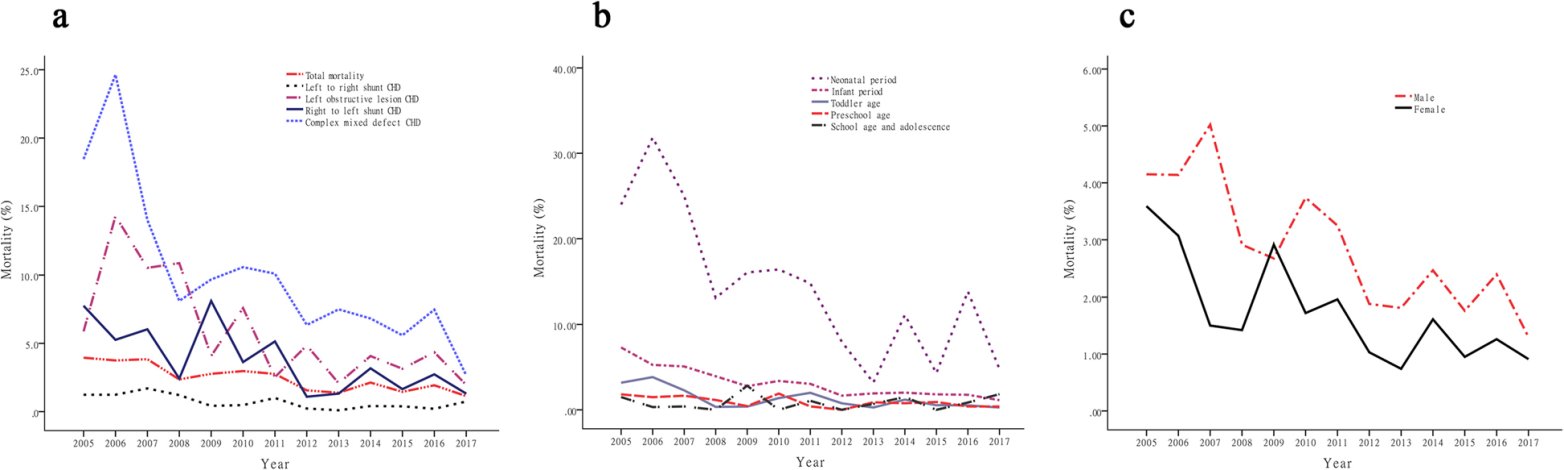
Distribution of CHD surgery mortality in children from 2005 to 2017. Chart **a** shows the mortality of different CHD disease types. Chart **b** shows the mortality of children at different ages. Chart **c** shows the mortality of CHD children by sex

The total number of deaths attributable to CHDs in children below 14 years old between 2005 and 2017 was 444 (325 (73.2%) males), while the total surgical volume was 19,144. The mortality of all children who underwent CHD surgery was 2.3%. Of the total mortality, 394 (88.7%) deaths occurred in children aged under 3 years. The mortality of CHD in all children fell from 3.9% in 2005 to 1.1% in 2017, and total mortality showed a gradual decline. Compared to 2005, the overall mortality rate decreased by 11.0% in 2017. The overall 13-year mortality of left to right shunt CHD, which fluctuated between 1.7% in 2007 and 0.1% in 2013, was 0.69%. The mortality of left obstructive lesion CHD was 4.5% for the 13-year study period, and it showed a gradual decline. It was the highest in 2006, up to 14.3%, and the lowest in 2017 (2.0%). For right to left shunt CHD, the total mortality at 13 years was 3.5%, and it gradually decreased. The 13-year fatality rate of right to left shunt CHD fluctuated between 7.8% in 2005 and 1.3% in 2017. The mortality of complex mixed defect CHD was highest in 2006 (24.6%) and lowest in 2017 (2.7%). The overall fatality rate was 8.6% and showed a downward trend in the volatility curve. Comparing these four types of CHD, complex mixed defect CHD had the highest mortality rate, left obstructive lesion CHD had the second, right to left shunt CHD had the third, and left to right shunt CHD had the lowest mortality rate (χ2 = 520.3, *P* < 0.05) (Fig. 3a and Table 2).

**Table 2:** Comparison of the impact of disease type, age and sex on the mortality

Different classifications		Death (N)	Alive (N)	Mortality (%)	z-test^*^ (Bonferroni method)	Chi-Square and ***P*** value
**Disease classification**	Left to right shunt CHD	82	11,731	0.69	a	χ2 = 520.3 *P* < 0.05
	Left obstructive lesion CHD	47	999	4.49	b	
	Right to left shunt CHD	146	4018	3.51	b	
	Complex mixed defect CHD	165	1748	8.63	c	
**Periods**	Neonatal period	129	931	12.17	a	χ2 = 529.3 *P* < 0.05
	Infant period	218	8229	2.58	b	
	Toddler age	47	3993	1.16	c	
	Preschool age	30	3162	0.94	c	
	School age and adolescence	20	2355	0.84	c	
**Male or female**	Male	325	11,426	2.77	a	χ2 = 26.4 *P* < 0.05
	Female	119	7244	1.62	b	

^*^ Each letter denotes a subset of Group categories whose column proportions do not differ significantly from each other at the .05 level

The change in the mortality data from 2005 to 2017 showed that the mortality rate of CHD surgery in children of different ages gradually decreased. The annual surgical volume during the neonatal period has increased year by year, from 25 cases in 2005 to 125 cases in 2017. A total of 1060 neonatal CHD surgeries were completed in the 13 years, with 129 deaths and a total mortality rate of 12.2%. For the neonatal period, the highest case fatality rate was 31.8% in 2006, and the lowest case fatality rate was 3.2% in 2013. The mortality rate of neonatal CHD surgery was significantly higher than that of other age groups in the past 13 years, but it generally showed a downward trend in the annual curve fluctuations (χ2 = 529.3, *P* < 0.05). The children with the most CHD surgeries at our hospital are infants. From 2005 to 2017, our center completed 8447 infant CHD operations; 218 of these children died, and the total fatality rate was 2.6%. From the data curve for the past 13 years, the overall mortality rate of CHD in infancy has gradually decreased, from 7.3% in 2005 to 1.1% in 2017. However, the annual surgical volume increased year by year, from 274 cases in 2005 to 975 cases in 2017, with 255.8% growth. For toddler age children, the total number of operations performed in the past 13 years was 4040; 47 of these children died, and the case fatality rate was 1.2%. The number of surgical cases each year also showed an increasing trend, from 221 cases in 2005 to more than 350 cases in more recent years. The mortality rate of preschool age children was relatively low, with a total CHD mortality rate of 0.9% (30/3192) over the past 13 years. The number of surgical cases per year was also relatively stable, with approximately 250 cases per year. The annual mortality rate also showed a downward trend, from 1.8% in 2005 to 0.4% in 2017. The 13-year total mortality rate in the school age and adolescent age group was 0.8%, which was the lowest among children of all age groups (χ2 = 529.3, *P* < 0.05). The number of surgical cases per year showed a gradual decline, from 313 cases in 2006 to 109 cases in 2017. Its total number of surgical cases at 13 years was 2375, which was the second least among all age groups; the surgical mortality rate in these cases fluctuated at approximately 0.8% (Fig. 3b and Table 2).

In both boys and girls, the annual mortality rate after CHD showed a gradual downward trend. The case fatality rate for boys fell from 4.2% in 2005 to 1.3% in 2017, and for girls, it fell from 3.6% in 2005 to 0.9% in 2017. Looking at the trends over the past 13 years, except for 2009, boys had a higher annual mortality rate than girls, which was also true for the comparison of the total mortality rate (χ2 = 26.4, *P* < 0.05) (Fig. 3c and Table 2).

## Discussions

Data from our heart center over the past 13 years showed that the overall in-hospital mortality rate for children with CHD was 2.3%, and it gradually decreased. Different types of heart disease have a significant impact on mortality. Complex mixed defect CHD and left obstructive lesion CHD have a high mortality rate, and left to right shunt CHD has the lowest mortality rate. The effect of different age stages on the mortality rate was also obvious. The lower the age, the higher the mortality rate. Neonatal CHD had the highest mortality rate, followed by infants. The mortality rate of CHD was also different in different sexes; it was significantly higher in males than in females. In general, surgery for CHD dramatically changed the outcomes for patients with even complex defects, and surgical mortality has significantly decreased over time. This trend is consistent with other reports [12–16].

At present, the heart research center at our hospital mainly treats simple congenital heart disease, such as ASD, VSD, and PDA, which is consistent with developed countries in Europe. However, the treatment effect for simple CHD is significantly better than that of developed European countries. Among them, the surgical mortality rate of ASD/VSD/PDA in our center is only 0.43%, compared with 0.99% in Europe [6]. However, the surgical mortality of complex congenital diseases such as PAT and left ventricular dysplasia is significantly higher in our center than that of developed European countries. For example [6], the mortality rates of Norwood surgery, TAPVC/PAPVC surgery, and PTA surgery in our center were 40.00, 21.74, and 1.95%, respectively, while the death rates for these surgeries in Europe during the same period were 24.01, 13.43, and 4.74%, respectively. The mortality rate for single ventricular surgery is 2.56% in China and 3.22% in Europe, while the mortality rate for left ventricular outflow tract surgery is 3.21% in China and 2.44% in Europe [6]. To improve the diagnosis and treatment of complex CHD, at least the following two aspects should cause concern [6]. First, by establishing a data platform for the entire life cycle diagnosis and rehabilitation of CHD, big data analysis and scientific research should be used to form a high-level norm to guide the treatment of CHD. Second, advanced technologies such as artificial intelligence, virtual reality, and 3D printing can be applied to achieve precise preoperative diagnosis and intraoperative navigation and improve the diagnosis and treatment of complex CHD.

The mortality rates after CHD surgery were similar in children over 2 years of age, while the mortality rate of infants under 1 year was significantly higher, especially in newborns, who had the highest mortality rate. Our data showed that the mortality rate of CHD surgery in newborns decreased year by year, which is similar to other reports worldwide. Andrzej Kansy and other scholars [17] have found similarities in the mortality rate of CHD in nearly 15,000 newborns in Europe. Newborns have relatively low body weight, require higher surgical skills, and have a higher risk of anesthesia; CHD surgery during the neonatal period is often a more difficult operation, and the requirements for perioperative management are higher. These factors [18] may be related to the relationship with mortality. Multivariate analysis [17] confirmed that lower body weight, higher basic Aristotle score, longer cardiopulmonary bypass time, longer aortic cross-clamp time, longer circulatory arrest time, and univentricular physiology were risk factors for hospital mortality. Some scholars [19] used the Japan Congenital Cardiovascular Surgery Database to find that congenital heart surgery (RACHS-1) score and categories have high discrimination power for predicting mortality.

It is puzzling that the mortality of CHD was different for different sexes. This pattern has not changed in our heart center for more than 10 years. Our research suggested that the case fatality rate in males was higher than that in females, which has been found in other studies [20]. There are many different opinions as to why this would occur [21–25], But the causes are not yet clear. The composition of heart disease in the different sexes, the age of patient at the time of surgery, and other factors that may affect this association need further analysis.

Our heart center team has continuously researched and challenged complex CHD, conquered CHD surgery for low-weight infants, improved surgical methods and techniques, and improved perioperative management [26–31]. And the treatment level has reached the international advanced level. This research showed that the annual amount of CHD in children was gradually increased, and the mortality rate has gradually decreased. It maybe is related to the surgical capabilities of pediatric cardiac surgeons, advances in extracorporeal circulation technology, increased levels of anesthesia, appropriate treatment during the perioperative period, and so on. In the past 13 years, the number of children’s cardiac surgery team and the size of the beds in our hospital have not changed much. However, in 2015, many doctors went abroad for further study, which affected the annual operation volume and the proportion of cyanotic CHD. This retrospective study explains the mortality and distribution of children’s CHD after thoracotomy, which helps us understand the prognostic factors of CHD. However, this study did not investigate the complications of CHD surgery and did not further study the composition and causes of death. This is an obvious deficiency of this study. More in-depth investigations are needed the future in the hope of finding correctable high-risk factors for mortality and morbidities or complications after cardiac surgery.

## Conclusions

Herein, we reported outcomes for all cardiac surgeries performed at our center in children younger than 14 years old with CHD over the past 13 years. The mortality rate of CHD surgery in children decreased year by year. The younger the age and the more complicated the cyanotic heart disease, the higher the mortality rate may be. Future challenges are the complex heart disease of low birth weight infants, the management of long-term complications after CHD, minimally invasive treatment technology, and the reduction of postoperative reoperation rates.

## Data Availability

All data generated or analysed during this study are included in this published article.
